# Correlation Study between Rural Human Settlement Health Factors: A Case Study of Xiangxi, China

**DOI:** 10.1155/2022/2484850

**Published:** 2022-05-12

**Authors:** Shuyuan Tong, Yafeng Zhu, Zhe Li

**Affiliations:** Department of Architecture, School of Architecture and Art, Central South University, Changsha 410075, China

## Abstract

With the emergence of the Industry 4.0 era in China, more refined methods are being proposed for healthy living requirements for human settlements. Since the rural human settlements in China are relatively backward, this study aimed to investigate the influencing factors of human health. First, through field surveys and questionnaires conducted with villagers in Xiangxi's traditional villages in Hunan Province, we analyzed the factors affecting human health qualitatively and quantitatively using the SPSS software. We identified three main dimensions affecting human health in rural human settlements including human behavioral activities, physical environment, and natural environment. Then, we used correlation analysis and multiple linear regression analysis methods to analyze the correlation between environmental factors and human health. The results showed that human activities, building physical environment, and natural environment are significantly correlated with human health. Among them, human behavior has the strongest correlation with health. This research contributes to creating healthy human settlements and guiding the creation of a healthy environment in rural China.

## 1. Introduction

Under the backdrop of the Industry 4.0 paradigm, various industries around the world are gradually intelligent, integrated, and automated. Big data, interconnections, and artificial intelligence have been applied in the construction elements, which tend to develop in the directions of digitized, multifunctional, and health promotion [1–10]. Compared with traditional construction methods, Industry 4.0 has brought an innovation orientation and technological advancements to China's construction industry and improved the quality of human settlements, efficiency, and safety of construction processes [11–17]. With the increased emphasis on health performance in settlements, how to build a living environment that meets the needs of human health in the era of Industry 4.0 remained unclear, especially on environmental factors that affect health in different settings. Existing research on healthy architecture and living environments is valuable and involves multidimensional and multilevel fields, such as medicine, psychology, society, and planning [18–21].

In recent years, under the background of Industry 4.0, science and technology have developed rapidly in China. Research on the health of human settlements has been extensive, but most of it relates to urban settlements, while studies on the construction of traditional villages have been rare [22]. The production mode of construction industrialization has many advantages, including high production efficiency, good quality, low construction cost, saving resources, and protecting the environment. It can greatly improve the problems existing in rural housing, with the following positive effects:

① Improve the quality of housing construction
② Improve energy conservation and emission reduction
③ Improve the style of rural housing
④ Improve the construction speed
⑤ Improve the comfort of living environment
⑥ Reduce construction costs
⑦ Stimulate rural economic growth and resolve excess capacity [23, 24]

Therefore, the industrialized production of construction is the development trend of the construction mode of new rural construction in the future, and the state has issued a series of policies and measures to promote the industrialized construction and development of rural areas. To further promote the steady and healthy development of industrialization in rural areas, the healthy living environment in rural areas [23, 24]isfocused. Due to the acceleration of China's economic, social development, and process of urbanization, a large number of young laborers from rural areas have poured into cities, resulting in the widespread phenomenon of elderly people and children left behind in rural areas and even the emergence of “ghost villages.” Numerous theoretical studies have shown that the living environment is directly related to the physical and mental health of the residents[25–27]. The rural environment in China is significantly different from the urban environment in terms of infrastructure, humanities and culture, and living conditions, thus resulting in significant differences in the physical and mental health levels of different groups of residents [28–30]. Generally, the village environment is rich in green resources and has good geography. In addition, the layout of the village and the housing structure follow the traditional idea of harmony between man and nature. These factors have a positive impact on human health. At the same time, bottlenecks in transportation, poor infrastructure construction, and physical environment need urgent improvements as such problems have a negative effect on people's physical and mental health. Therefore, to optimize rural human settlements and improve their physical and mental health, we aimed to conduct a correlation study on the influencing factors of health among rural humans.

## 2. Literature Review

Human health is a complex and comprehensive research topic involving multiple fields of study, such as medicine, sociology, and environmental science. Human settlement is a complex manifestation of many factors related to humanity, nature, and society. A healthy and comfortable human settlement can stabilize people's emotions and is conducive to physical and mental health; hence, building healthy human settlements is one of the trends of the construction industry under the background of Industry 4.0. In foreign countries, they have paid more attention to the construction of healthy human settlements earlier and have obtained substantial research results. Many international organizations, such as the World Health Organization, the European Environment Agency, and the United Nations Environment Program, have issued a series of guidelines and standards for healthy environments [31, 32]. In recent years, the WELL building standards released in the United States have established indicators for assessing the building environment across seven areas, namely air, water, nutrition, light, fitness, comfort, and mood. The associated research has presented various perspectives [33]. Previous research scholars have used different methodologies and techniques to systematically assess the health performance of living environments involving different spatial environments and specific populations [34, 35]. In addition, previous mixed-methods studies have examined the interactions and feedback between multiple environmental physical factors and human health and propounded the possibilities and ways to achieve healthy living in an increasingly urbanized built environment [25, 36–44].

With the emphasis on healthy human settlements in China, deep and relevant research in multiple fields has been developed. Numerous scholars have analyzed in detail the health indicator systems of different urban environments, including transportation systems, infrastructure, air quality, sewage treatment, health care, social development, environmental management, and lifestyles. Many analysis methods have also been used to conduct relevant health assessments on environmental factors [45–52]. Most studies on healthy human settlements in China have focused on urban areas, whereas the rural environment in China has significant differences from urban areas in terms of spatial distribution, lifestyle, humanistic characteristics, infrastructure, and other environmental factors. Many studies have shown that various environmental factors with distinct rural characteristics influence people's lifestyles and behaviors at the social, humanistic, and material levels, which in turn have positive or negative effects on human physical and mental health [53–58]. Therefore, this work took Xiangxi's traditional villages in China as an example and conducted a correlation study on their human settlements related to human health.

## 3. Research Plan Design

### 3.1. Research Object

**Figure 1:**
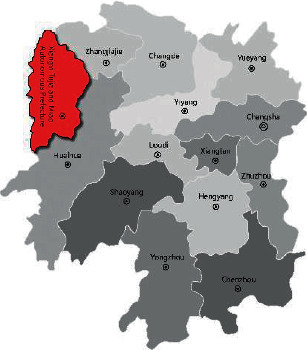
Area where the Xiangxi villages are located.

There are many traditional Chinese villages with obvious and different characteristics. With regard to the list of five batches of “Chinese traditional villages” published, the number of traditional villages distributed in Xiangxi has reached 172. At the same time, due to the special geographical and ecological environment and historical changes, Xiangxi still retains a relatively complete village form and humanistic tradition. Since ancient times, many ethnic groups, including Miao, Han, Tujia, Dong, and Yao, have been living in the area, with strong ethnic characteristics and living features. Therefore, we selected traditional villages with essentially complete settlement patterns as the main research focus. Our list comprised Lahao Village in the Duli Township of Fenghuang County, Laodong Village in the Machong Township, and Guantianshan Village (Figure 1). The long-term residents of the village were randomly selected as the study participants. Following uniform standards, we adopted methods such as the interview and participatory observation method to obtain information from the participants. Basic data were obtained by mapping and basic testing the human settlements of the villages.

### 3.2. Research Methods

To obtain the health indicators of the permanent residents in the Xiangxi rural environment, this study used the SF-36 scale to measure the eight dimensions of general health, physical function, physical role, somatic pain, vitality, social function, emotional function, and mental health [59, 60]. The average of the total scores of the eight dimensions was used as the dependent variable *y* as an overall indicator for health evaluation. Second, according to the survey indicators of the human settlements, a questionnaire for the healthy villages' human settlements was constructed by reviewing the literature and consulting experts while considering the operability of data collection and the specificity of village lifestyles. When screening the evaluation indicators of the questionnaires, the first-level criteria layer was determined as the three dimensions of human behavior, natural environment, and physical environment by literature review. The secondary evaluation factors were 16 relevant environmental evaluation indicators based on existing studies, and after consulting with relevant scholars and experts by email in various fields such as architecture, planning and design, and landscape professionals, the evaluation feedback was filtered and modified based on expert feedback. After multiple additions and deletions of evaluation factors, a total of 18 factors were determined from three dimensions of human behavior, natural environment, and building physical environment. Related questions were set up around the 18 factors to form a structured questionnaire (Table 1). By designing a Likert scale, this study divided the evaluation factors of the environment into five evaluations based on the statements regarding the attitudes of approval or disapproval: very good, good, fair, poor, and very poor. We transformed the scale into a fixed-distance evaluation level of subjective evaluation [61, 62].

Correlation analysis was performed on the health self-assessment results and environmental influences. Subsequently, multiple linear regression analysis was used to establish regression equations to further explore the association between health and environment. All analyses were performed using SPSS.

### 3.3. Research and Analysis

#### 3.3.1. Sample Characteristics

Of the 160 questionnaires distributed, 153 participants completed the assessment. The effective response rate was 95.6%. Among the 153 qualified participants, 73 (47.7%) were aged over 60 years and 83 (54.2%) were female (Table 2). In the SF-36 health assessment, 19 (12.4%) rated their overall health status as “excellent,” 53 (34.6%) as “very good,” 45 (29.4%) as “good,” 35 (22.9%) as “fair,” and 1 (0.7%) as “poor.”

Xiangxi is in the northwestern part of Hunan Province, China, east of the Wuling Mountains areas of the Yungui Plateau. In total, 401 villages in Hunan Province are included in the fifth batch of the list of traditional villages in China, and Xiangxi accounts for 90 villages. Xiangxi is dotted with many Miao traditional villages of different forms and long histories. The climate in Xiangxi has four distinct seasons, with rainfall concentrated between April and June, accounting for 41–47% of the annual precipitation. Due to the complex terrain and the influence of airflow, the amount of solar radiation received varies, and the lighting and temperature of each region are different.

**Figure 2:**
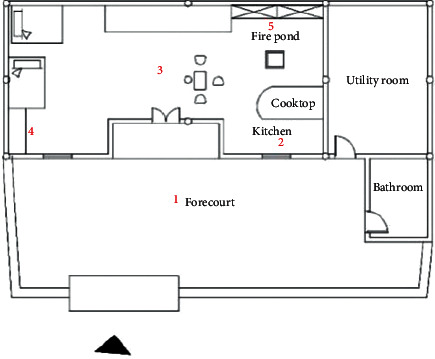
Temperature, humidity, and illuminance detection point of a typical traditional house.

As an example, a typical local traditional residential building was mapped and tested for temperature, humidity, and light level. Using a French KIMO HD100S portable hygrometer thermometer with a temperature and humidity monitor, measurements were taken during the hours of 8 : 00–19 : 00 from January 15 to January 17 in a typical local winter season and from July 9 to 11 in a summer season, and the temperature and humidity of the measurement points (at 1.1 m from the ground) were recorded with an interval of 30 minutes. The measurement points were at five locations: outdoor courtyard, lobby, bedroom, kitchen, and fire pit, as shown in Figure 2 and Table 3. The results showed that the indoor and outdoor humidity was generally high, and the difference between the indoor and outdoor temperatures in summer and winter was within 3 °C due to the influence of the houses' construction materials and methods. The residential heating in winter mainly relied on firewood, which causes pollution.

To measure the indoor and outdoor illumination conditions, a Sigma AS82 handheld illuminance meter was used, and measurements were made at 12 : 00 noon on January 15 in winter and July 9 in summer. The weather we chose to measure was cloudy days without precipitation in winter and sunny days in summer, as shown in Table 4. Considering the different degrees of the influence of illumination on functional rooms, the measurement points included the outdoor courtyard, foyer, bedroom, kitchen, and fire pit. In addition to the five measurement points, the bathroom was also included as an illumination measurement point, as shown in Table 4. Due to the long scale of building depth and the limitation of building materials for the window and door openings, the indoor lighting was fair.

#### 3.3.2. Reliability and Validity Test

This study used Cronbach's alpha coefficient to test the inherent reliability of the survey questionnaire. The range of Cronbach's alpha coefficient is between 0.00 and 1.00, with higher values indicating higher reliability and lower values indicating lower reliability. The survey results show that the overall Cronbach's alpha coefficient of the dimension of human behavior in the questionnaire was 0.723; the coefficient of the second dimension of the questionnaire of the physical environment of the building was 0.972; and the coefficient of the third dimension of the natural environment was 0.849, indicating that the questionnaire had good reliability and the measurement results were reliable (Table 5).

Validity tests were conducted using structural validity, and the KMO test and the Bartlett sphere test were used. When the KMO test coefficient is greater than 0.50 and the Bartlett sphere test, X2 has a *P* value less than 0.05, and the research questionnaire has structural validity. Validity tests were performed on the three dimensions, and the respective KMO test values were 0.702, 0.903, and 0.801, all of which were greater than 0.70. The corresponding *P* values were less than 0.05. The significant difference between the correlation coefficient and the unit matrix indicates that there is a correlation among the survey data. Therefore, the questionnaire has structural validity.

#### 3.3.3. Correlation Analysis

Through the analysis of the questionnaire, the total score of the eight dimensions of the SF-36 health evaluation result was used as the dependent variable *y*, and the 18 environmental factor assignment calculations were divided into three dimensions using SPSS for correlation analysis. The Pearson correlation value is between [−1, +1]. A value greater than 0 means positive correlation, and a value less than 0 means negative correlation. The closer the absolute value is to 1, the stronger the correlation is. Since the data belonged to different populations, to decrease potential confounding of health status, gender, age, and education level were added to the correlation analysis as independent variables. In the correlation analysis results, the significance of the four variables of people, build, nature, and age is 0.000, 0.039, 0.009, and 0.000, all of which were less than 0.05 and statistically significant. Among population attributes, gender, education level, and health status had no significant correlation, while age and health status were significantly negatively correlated. In addition, the Pearson value corresponding to age is -0.495, indicating a negative correlation, and the older the age, the worse the health. In the three dimensions of people, build, and nature, the value of the Pearson correlation with *y* was 0.520, 0.167, and 0.211, all of which are positive numbers, suggesting that the variables of the three dimensions are positively correlated with the dependent variable *y*.  Table 6 shows that human behavior has a stronger correlation with health status, whereas the correlation between the physical and natural environment of the building and health status is weak. Among these three dimensions, the factors that have the strongest correlation with health status are “daily physical activity level,” “summer indoor temperature evaluation,” and “housing orientation evaluation.”

#### 3.3.4. Regression Model Establishment

The independent variables were quantitative and more than one in number in our research, so we choose the multiple linear regression analysis method to examine the relationship between the independent variables of the three dimensions and health status. The mathematical model of multiple linear regression is *E*(*y*) = *β*_0_ + *β*_1_*x*_1_ + *β*_2_*x*_2_ + … + *β*_p_, where *x*_p_ explains the linear change in *y* caused by the change in variable *x*; *x*_p_(*i* = 1,…, p) is the independent variable; *p* is the number of independent variables; *β*_0_ is the constant term of the regression equation; and *β*_p_(*i* = 1,…, p) is the regression coefficient.

The scores of gender, age, education level, mean values of the scores of human behavior, building physical environment, and natural environment were taken as the six independent variables, and regression analysis was performed with the dependent variable as health status. The results show that the D-W test value was 1.630 and its value tended to 2, indicating that there was no autocorrelation. That is, heteroscedasticity was considered not to exist (Table 7). *R*^2^ was 0.508, which suggested that the independent variables of the three dimensions could jointly explain 50.8% of the dependent variable.

We performed the F test on the model, and the model's *P* value is less than 0.001, indicating that the model passed the F test, and the linear relationship was valid. At the same time, the statistical significance of the regression model also indicated that compared with the null model, the inclusion of independent variables helped to predict the dependent variable, or that the model was better than the null model; that is, at least one of the six independent variables could affect the health status (Table 8).

**Figure 3:**
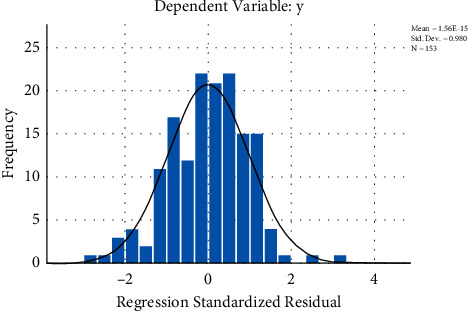
Histogram residual.

**Figure 4:**
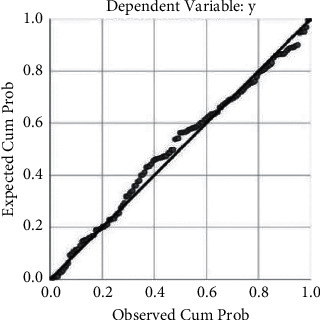
Normal P-P plot of regression standardized.

Through the verification analysis, the results of the histogram showed that the curve was roughly normal distribution; the closer the distribution of each point in the normal P-P graph was to the diagonal line, the closer the data were to the normal distribution. Figures 3 and 4 depict that the residuals were approximately normally distributed, and the regression model was statistically significant.

In the regression, the dependent variable *y* was health status; the independent variables were gender, age, education level, people, building, and nature, which represented the four independent variables of age, human behavioral activities, building physical environment, and natural environment, respectively. The *P* value for each of these four independent variables was less than 0.05. The following equation was established:

(1) $$\begin{array}{c} Y=122.422-\text{gender}*2.230-\text{age}*10.395-1.312*\text{ education level }+\text{ people }*\;12.095\;+\text{ building }*\;4.259\;+\text{ nature}*5.122. \end{array}$$

That is, for each additional person, *y* increased by 12.095 units; for each unit increase in building, *y* increased by 4.259 units; for each unit volume increase in nature, *y* increased by 5.122 units; and for every unit increase in age, *y* decreased by 10.395 units (Table 9).

## 4. Conclusions and Recommendations

With the acceleration of China's economic and social development and urbanization, the population structure of China is gradually changing into an aging population. In this study, 47.7% of the study participants were aged 60 years and above; most of the elderly were unaccompanied by children at home; and their physical functions have reduced over time. Some studies have shown that age is one of the main elements affecting health assessment, followed by gender, income, physical environment, health-related behaviors, and regional medical level. These are all factors relevant to health status [63–67].

The results of the univariate analysis in this study show that age was correlated with overall health negatively and significantly; that is, the higher the age, the lower the health self-assessment score. Income and education were not significantly correlated with health assessment results, and this result was related to the overall low income and low education level of the research subjects. At the same time, the results of this survey show that gender was not significantly correlated with health assessment results, because within the scope of the survey, gender differences are associated with cultural, social status, division of labor, living habits, etc., all of which can indirectly affect physical health [68, 69]. Among the eight dimensions of health assessment, older adults had lower mental health scores and generally were depressed and had negative emotional conditions due to lack of family companionship and recreational life. Some scholars have found that gender, age, ethnicity, hobbies, life satisfaction, and physical health were associated with mental health among the Xiangxi older adults [70–72].

Second, about 82.4% of the villagers among the research subjects needed to perform daily work activities and therefore maintained a certain amount of daily exercise. In the correlation analysis of this study, the factor “daily physical activity level” in the dimension of “human behavior” was significantly correlated with the health status. In the specific environment and cultural background of rural areas in China, work activity is an essential life support behavior, and this necessary lifestyle has a positive impact on the health of the research subjects. In the survey area, many medical records and surveys have shown that the lifestyle, medical condition, and labor intensity had a certain impact on human physical and mental health, and the health level of urban and rural residents is different [73–75].

The building physical environment has a direct impact on human perception, and the results of this study show that the “building physical environment” has a significant correlation with health status. We investigated the issues related to the setting of room temperature and humidity, kitchen drainage, and room spatial scale in the building physical environment. The strongest correlation with the dependent variable health status was the “summer indoor temperature evaluation.” This is consistent with many existing research results such as indoor air pollution, heat, and humidity conditions affecting human health [76].

The natural environment is closely related to human health. In traditional Chinese culture, related theories emphasize the harmonious relationship between nature, architecture, and human beings. Our findings suggested that the natural environment and health status have a significant correlation, among which the univariate analysis showed that the “housing orientation evaluation” under the “natural environment” dimension had the strongest correlation [77–80].

Lastly, we proposed the following suggestions for healthy rural human settlements in Xiangxi. (1) Pay attention to the physical and mental health of the elderly living in the villages; increase the village infrastructure for adapting to the aged; improve the medical service system; enrich the recreational life of the middle-aged and elderly; and realize “healthy elderly care.” At the same time, promote healthy lifestyles and popularize knowledge about healthy living for all. (2) Improve the living environment of villages and improve the comfort and convenience of the indoor environment of buildings. Modernize and renovate the pollution sources of the indoor environment and pay attention to the heat insulation performance of the houses. (3) Consider the orientation and the influence of light in the layout and site selection of the building. Draw lessons from the traditional village Taoist law of nature, the harmony between man and nature, and the movement according to time. Focus on the relationship between man and nature in urban architectural design to promote a positive impact on human health.

The questionnaire design in this study considered limited dimensions and factors. Considering the operability and practicality of the data, it mainly focused on architecture and environmental psychology, but many environmental impact factors were not included. Future studies should consider including more covariates.

## Figures and Tables

**Table 1:** Human settlement assessment factors of Xiangxi villages.

Dimension (A)	Evaluation factor (B)
Human behavior (A1)	B1 daily physical pattern and frequency
	B2 daily walking distance
	B3 frequency of communication with neighbors
	B4 daily hobbies and interests
	B5 daily cooking style
	B6 understanding of and access methods to health knowledge
Natural environment (A2)	B7 air quality of the natural environment
	B8 climate temperature and humidity comfort
	B9 daily noise pollution
	B10 waste disposal methods
	B11 medical level and care
	B12 living house orientation
Physical environment (A3)	B13 indoor temperature and humidity in summer and winter
	B14 construction methods and materials of the houses
	B15 indoor hygienic environment conditions
	B16 indoor ventilation and lighting conditions
	B17 indoor and outdoor vegetation and greenery
	B18 embodiment of regional culture decoration

**Table 2:** Summary of basic information of the questionnaire.

Statistical index		Frequency	Proportion (%)
Gender	Male	70	45.8
	Female	83	54.2
Age	≤18	8	5.2
	19–39	27	17.6
	40–59	45	29.4
	60–79	65	42.5
	≥80	8	5.2
Education level	Primary school and below	116	75.8
	Middle school	23	15.0
	High school/junior college	12	7.8
	College and above	2	1.3

**Table 3:** Temperature and humidity data of the measurement points in summer and winter.

Measurement point	Average summer temperature (°C)	Average summer humidity (%)
1	31.5	82.7
2	29.6	88.5
3	29.4	85.7
4	29.8	88.9
5	29.1	89.2

1	6.4	75.7
2	7.3	78.5
3	7.1	77.7
4	7.9	79.6
5	8.5	76.9

**Table 4:** Illuminance data of the measurement points in summer and winter.

Measurement point	Summer illuminance (lux)	Winter illuminance (lux)
1	63701	3614
2	330	281
3	236	168
4	305	204
5	179	115
6	158	177

**Table 5:** Reliability test results.

Cronbach's coefficient	Number of terms
People 0.723	4
Building 0.972	10
Nature 0.849	4

**Table 6:** Correlation analysis results.

		Gender	Age	Education level	People	Building	Nature	*Y*
Gender	Pearson's correlation	1	0.062	0.015	0.149	−0.054	−0.050	−0.012
	P (two-tailed)		0.448	0.859	0.067	0.508	0.542	0.882
	N	153	153	153	153	153	153	153

Age	Pearson's correlation	0.062	1	−0.355^*∗∗*^	−0.140	−0.078	−0.235^*∗∗*^	−0.495^*∗∗*^
	P (two-tailed)	0.448		0.000	0.084	0.340	0.004	0.000
	N	153	153	153	153	153	153	153

Education level	Pearson's correlation	0.015	−0.355^*∗∗*^	1	−0.009	−0.025	0.059	0.102
	P (two-tailed)	.859	0.000		0.913	0.758	0.470	0.211
	N	153	153	153	153	153	153	153

People	Pearson's correlation	0.149	−0.140	−0.009	1	−0.116	−**0**.012	0.520^*∗∗*^
	P (two-tailed)	0.067	0.084	0.913		0.154	0.887	0.000
	N	153	153	153	153	153	153	153

Building	Pearson's correlation	−0.054	−0.078	−0.025	−0.116	1	−0.056	0.167^*∗*^
	P (two-tailed)	0.508	0.340	−0.758	0.154		0.489	0.039
	N	153	153	153	153	153	153	153

Nature	Pearson's correlation	−0.050	−0.235^*∗∗*^	0.059	−0.012	−0.056	1	0.211^*∗∗*^
	P (two-tailed)	0.542	0.004	0.470	0.887	0.489		0.009
	N	153	153	153	153	153	153	153

Y	Pearson's correlation	−0.012	−0.495^*∗∗*^	0.102	0.520^*∗∗*^	0.167^*∗*^	0.211^*∗∗*^	1
	P (two-tailed)	0.882	0.000	0.211	0.000	0.039	0.009	
	N	153	153	153	153	153	153	153

^*∗∗*^ *P* < 0.01. ^*∗*^*P* < 0.05.

**Table 7:** D-W test results.

Model	R	*R* ^2^	Adjusted *R*^2^	Error in standard estimate	Durbin–Watson
1	0.713^a^	0.508	0.488	18.87590	1.630

a. Predictor variables: constant, nature, people, building, gender, age, and education level. b. Dependent variable: *y*.

**Table 8:** *F* test results.

Model		Sum of squares	Degree of freedom	Mean square	F	P
1	Regression	53792.997	6	8965.500	25.163	0.000^(b)^
	Residual	52019.735	146	356.300		
	Sum	105812.733	152			

(a) Dependent variables: y. (b) Predictor variables: constant, nature, people, building, gender, age, and education level.

**Table 9:** Regression analysis results.

Model	Nonstandardized coefficient		Standardized coefficient	*t*	*P*
	B	Standard error	*β*		
Constant	122.422	12.203		10.032	.000
Gender	−2.320	3.115	0-.044	−0.745	.458
Age	−10.395	1.757	0-.387	−5.917	.000
Education level	−1.312	2.417	0-.034	−0.543	.588
People	12.095	1.464	0.496	8.259	.000
Building	4.259	1.266	0.199	3.363	.001
Nature	5.122	2.240	0.137	2.286	.024

Dependent variable: *y*.

## Data Availability

The data used to support the findings of this study are included within the article.
